# Digital Covid Certificates as Immunity Passports: An Analysis of Their Main Ethical, Legal, and Social Issues

**DOI:** 10.1007/s11673-022-10209-4

**Published:** 2022-09-19

**Authors:** Íñigo de Miguel Beriain, Jon Rueda

**Affiliations:** 1 Department of Public Law, University of Basque Country, Leioa, Spain; 2 Department of Philosophy 1, University of Granada, Granada, Spain

**Keywords:** COVID-19, Digital Green Certificate, Global fairness, Immunity passports, Public health ethics

## Abstract

Digital COVID certificates are a novel public health policy to tackle the COVID-19 pandemic. These immunity certificates aim to incentivize vaccination and to deny international travel or access to essential spaces to those who are unable to prove that they are not infectious. In this article, we start by describing immunity certificates and highlighting their differences from vaccination certificates. Then, we focus on the ethical, legal, and social issues involved in their use, namely autonomy and consent, data protection, equity, and international mobility from a global fairness perspective. The main conclusion of our analysis is that digital COVID certificates are only acceptable if they meet certain conditions: that they should not process personal data beyond what is strictly necessary for the aimed goals, that equal access to them should be guaranteed, and that they should not restrict people’s autonomy to access places where contagion is unlikely. We conclude that, if such conditions are guaranteed, digital COVID certificates could contribute to mitigating some of the most severe socioeconomic consequences of the pandemic.

## Introduction

On March 3, 2021, the president of the European Commission, Ursula von der Leyen, unveiled that the commission planned to create an immunity certificate in an effort to restore travel for business and tourism. Soon afterward, on June 14, “Regulation (EU) 2021/953 of the European Parliament and of the Council of 14 June 2021 on a framework for the issuance, verification and acceptance of interoperable COVID-19 vaccination, test and recovery certificates (EU Digital COVID Certificate) to facilitate free movement during the COVID-19 pandemic” was approved (European Commission 2021a). It was based on four main points:

- It covered three types of certificates—vaccination certificates, test certificates (NAAT/RT-PCR test or a rapid antigen test), and certificates for persons who have recovered from COVID-19.
- Certificates should be issued in digital form or on paper. Both will have a QR code containing the necessary key information as well as a digital signature to make sure the certificate is authentic.
- The commission would build a gateway and support member states to develop software that authorities can use to verify all certificate signatures across the European Union. No personal data of the certificate holders would pass through the gateway or be retained by the verifying member state.
- Certificates would be available free of charge in the official language or languages of the issuing member state as well as in English. (European Commission 2021b).

On this basis, on July 1, 2021, the Digital COVID Certificate was implemented throughout the European Union and it has been used in international transport since then. Moreover, some countries, such as France, Italy, and Spain, have used this tool to incentivize vaccination or to protect some concrete spaces—restaurants, concert halls, gyms, and so on—by obliging organizers or owners to ask attendants for their immunity certificates as a requirement to gain access to their facilities.

Under such circumstances, we might have some good reasons to be optimistic about the possibility that a novel and powerful weapon has been added to the arsenal against the pandemic (de Miguel Beriain 2020). However, such certificates also brought a lot of ethical, legal, and social issues. This paper aims to perform an urgent analysis of such issues. For this purpose, we start by exposing the differences between the aforementioned tool and the “immunity passports” that were strongly criticized by parts of the bioethics community. Then, we focus on some concrete burning issues, such as discrimination, consent, data protection, equity, and global fairness. Of course, one could simply oppose immunity certificates on a scientific basis, by stating that up until this point the tools aimed at measuring immunity are not efficient (Word Health Organization [WHO] 2020a) or by considering that vaccines do not provide any kind of sterilizing immunity (see below). However, since this is not a strict ethical, legal, or social issue, but a scientific topic that would deserve a separate paper devoted to it, we will leave it aside and work on the supposition that such certainty does exist.

Thus, in this paper, we assume that we can test immunity and that vaccines reduce infectiveness significantly. We argue that certificates should be modified, so that different combinations of immunity-providing situations (such as one dose + contagion, for instance) could serve to obtain the certificate. On the other hand, if vaccines do not provide significant sterilizing immunity, they should not be part of an immunity certificate. Instead, only a negative response to a test or immunity provided by contagion should serve to obtain it. If these conditions do not apply, immunity certificates could hardly be considered efficient tools to prevent the spread of the virus.

## Are Immunity Certificates Similar to Vaccination Certificates? A Preliminary Conceptual Clarification

Before debating the ethics of immunity certificates, we must clarify the confusing terminology that hinders the debate. Immunity certificates have been defined as “digital or physical documents that certify an individual has been infected and is purportedly immune to SARS-CoV-2” (Phelan 2020). This is a poor description since it does not clarify the type of immunization gained by the individual, and this is a key question. There are two different kinds of immunity. Functional immunity occurs when even though a body recognizes a pathogen and impedes the disease, it cannot avoid transmitting the virus. Thus, people with functional immunity can infect other people (Advisory Board 2020). On the other hand, sterilizing immunity is “a unique immune status, which prevents effective virus infection in the host” (Dutta et al. 2016). This second kind of immunity prevents us from infecting others.

The concept of immunity passports should be linked to the “level of threat” posed by their owner, that is, their capability (or lack of) to spread the virus. Therefore, a person should only obtain a certificate if they are not contagious or less likely to be contagious than someone who is not holding such a certificate. This only happens if they are not infected or if they have gained at least a significant sterilizing immunity. This is why immunity certificates should be considered a “risk-free certificate” (WHO 2020a).

Keeping this in mind, it is clear that immunity certificates are different from vaccination certificates, which might be defined as documents that provide evidence that someone has been vaccinated against a concrete virus. For instance, the WHO already uses an established and trustworthy international system of certification for diseases such as yellow fever (WHO 2020b; Vanderslott and Marks 2021). Unlike an immunity certificate, a vaccine certification does not refer to the immunological status of an individual, but to their vaccination record. This is why they cannot be obtained by providing evidence of having recovered from COVID-19 or by recently testing negative for the virus. In that sense, vaccination certificates are a more deficient tool to reduce the spread of a virus (since vaccination at the moment does not ensure gaining sterilizing immunity) and are less equity-friendly tools, since they discriminate against all those who have been unable to gain access to vaccination or, simply, opted out from vaccination processes. Thus, immunity certificates are a wider, more flexible tool, which allows different ways to prove that someone will not probably spread a virus, instead of only certifying that they have been vaccinated.

Last but not least, one should better consider immunity certificates as a flexible tool. Similar to aviation licensing requirements, obtaining immunity certificates could be made even more stringent when applied to specific, high-risk activities, such as working in a nursing home, and could permit exceptions and gradations (Persad and Emanuel 2020). For instance, a certificate that could provide access to a sports competition in the open air should be invalid to enter a nightclub, since the conditions of both types of activities are considerably different. Similarly, some certificates should depend on the concrete type of vaccine administrated (or the number of doses) or the type of test performed by the individual, since they provide different levels of sterilizing immunity or certainty of not being infected.

## Discrimination

One of the main reasons to oppose immunity certificates is that they might discriminate unfairly against non-holders, a situation that would not only be unethical (Baylis and Kofler 2020; Phelan 2020), but clearly against binding legal clauses, such as article 8 of the European Convention of Human Rights (European Court of Human Rights and Council of Europe 1950). Indeed, there are intuitive reasons to think that immunity certificates might provoke a discriminatory situation. If vaccines are not widely accessible and if tests are not affordable for everyone, it is perfectly logical to consider that underprivileged people would have far greater difficulty in obtaining a certificate.

This objection is twofold. On the one hand, it is related to the fact that not everyone has the means to provide evidence of sterilizing immunity. Thus, if certificates are not accessible to all in a fair way, the whole system would be unacceptable. However, since this concern is mainly related to equity, we will return to it later in the last section. On the other hand, the appeal against discrimination might be grounded in the idea that differentiating between people based on their immunological status would be discriminatory by nature. This interpretation, in our opinion, is misleading. There are no compelling arguments to accept that characterization. Public health policies have traditionally treated people in different ways according to their immunological status. This is the reason why those who are infected are isolated from the rest of the people. Therefore, immunity certificates could be acceptable if they only distinguish between people on the possibility of being contagious. Indeed, this is perfectly in line with the Convention for the Protection of Human Rights and Fundamental Freedoms, signed in Rome on November 4, 1950. This is a fundamental legal framework that defends individual rights and freedoms in all signatory countries, including most European countries. According to article 5, “Everyone has the right to liberty and security of person. No one shall be deprived of his liberty save in the following cases and in accordance with a procedure prescribed by law: (…) (*e*) the lawful detention of persons for the prevention of the spreading of infectious diseases” (European Court of Human Rights and Council of Europe 1950). Impeding the access of infectious people to particular spaces could be a necessary policy to prevent the spreading of infectious diseases.

Let us consider an example that could serve to reinforce our position. If we were facing an Ebola pandemic and someone was bleeding in a queue to access a theatre, preventing this person from entering may be justified. It would be imprudent and even unethical to let them share the space with non-infected people. Thus, the focus should not be placed on whether impeding a person from travelling or gaining access to some concrete facilities is discriminatory or not, but on the tools that could be used to determine whether such a person is infectious or not. Since Aristotle, a core tenet of justice ethics is to treat like cases alike. When substantial differences between immunity statuses exist, it could be wrong *not* to differentiate (Hall and Studdert 2020; de Miguel Beriain and Rueda 2020). Being a carrier (actual or potential) of a serious contagious disease is an ethically significant difference to justify dissimilar treatment during a pandemic. Therefore, immunity certificates could hardly be considered discriminatory tools in an ethically relevant sense. That said, we will now discuss autonomy and consent issues.

## Autonomy and Consent

An additional argument against immunity certificates is that they could threaten personal autonomy. In fact, although one might assume that those who obtained these certificates would have given their consent for the tests capable of ensuring their sterility, the truth is that such consent would be conditioned. Consent can only be valid if it is freely given. If certificates were to become a necessary requirement to access multiple public or private spaces, the consent would no longer be free, but forced, and therefore autonomy would suffer a serious restriction.

This argument is certainly convincing: certificates diminish autonomy. However, it does not really prevent us from concluding that, on this basis, we should ban their use. Indeed, in pandemics, it often happens that we cannot choose between a bad and a good option, but between a bad and a worse option. In terms of autonomy, certificates are clearly a restrictive tool if you compare them with the type of individual freedom we enjoy in our ordinary lives. However, if we analyse the situation during a pandemic, one might argue that immunity certificates could even increase autonomy. To understand why, one should start by thinking that, during the most severe periods of the pandemic, many common freedoms were seriously restricted due to public health reasons. This means that most people lost fundamental rights and freedoms, even though they did not pose any threats to public health. If we could significantly increase the safety of spaces such as workplaces, medium and long-distance transport systems, or leisure facilities, this could help us avoid general lockdowns or unnecessary quarantines. Thus, people unable to spread the virus would be clearly gaining autonomy. Moreover, even those people who should isolate due to direct contact with an infected person could recuperate some autonomy, since they could be allowed to leave home, provided that they do not access a secured space. Therefore, one can hardly sustain that immunity certificates reduce our autonomy. Rather, they could reverse some restrictions that curtail basic freedoms.

To sum up, immunity certificates work well with the idea of autonomy since they allow people who do not entail a threat to public health to enjoy a normal life, a situation that could hardly happen without the use of such certificates. Our refutation is consistent with the argument elaborated by Persad and Emmanuel:

People must be allowed to pursue their life plans unless doing so is incompatible with public health. The least restrictive alternative principle supports using COVID-19 immunity licenses if available. Current liberty-limiting restrictions on gatherings, work, and travel are justified because infected people may be harmed or die and may harm others by spreading disease or overburdening hospitals. But they are not justified when applied to people at little or no risk of infection. The principle of the least restrictive alternative supports giving people a chance to show that they are immune. (Persad and Emanuel 2020, 2241)

## Data Protection

Immunity certificates have been criticized on the basis that they erode privacy (Kofler and Baylis 2020). Indeed, personal data protection issues are extremely relevant when evaluating the morality of immunity certificates since they will contain special category data regarding health. Therefore, it could only be processed if certain circumstances apply. In the European Union arena, this has been adequately ensured by the available regulations. Indeed, data processing is only lawful if the circumstances described in Article 9.2 of the General Data Protection Regulation (GDPR) of the European Union and the concurrence of one of the legitimation bases provided for in Article 6 of the same legislation (European Parliament and the Council of the European Union 2016) apply. Thus, provided that the use of this system is rendered necessary and proportionate to combat COVID-19, since any less intrusive alternative method is not available, it is possible to find a way to conciliate data protection and public health goals.

However, how could this equilibrium between data protection issues and COVID-19 mitigation be reached in the case of immunity certificates? In our opinion, we should start by understanding the conditions under which such processing could be considered lawful. For instance, article 9.2 (*i*) of the GDPR allows the processing of special categories of personal data if it is deemed necessary for public health by the European Union or by member state law. This law provides suitable and specific measures to safeguard the rights and freedoms of the data subject, in particular, professional secrecy. Furthermore, principles such as data minimization or storage limitation should play a key role on this issue: only data that are strictly necessary to provide (or deny) an immunity certificate should be processed and only during the minimum time needed. Therefore, the implementation of immunity certificates would probably require the elaboration of a new legal framework, including concrete measures to protect the rights and freedoms of the data subject. They should be put in place by design and default in order to counter or mitigate such risks. Once such safeguards were provided, the processing of data would be lawful under the condition settled by article 6.1 € of the GDPR: that it would be necessary “for the performance of a task carried out in the public interest or in the exercise of official authority vested in the controller” (European Parliament and the Council of the European Union 2016).

Thus, the response to the objection is quite simple: privacy is not a good reason to oppose immunity certifications if adequate safeguards are adopted to ensure that data processing does not provoke unethical consequences. These include the following (Ní Loideain 2020): (1) whether, in addition to the person’s health data, other data will be used (e.g., biometric data used to verify and authenticate an individual when entering a building); (2) what authorities will be given the power to collect and request access to the passport, and for what purpose; (3) whether the data will be shared with other bodies, and for what purposes; (4) what independent oversight body will be responsible for the monitoring and review of the process, and so on. The safeguards introduced by the GDPR could be considered as the golden standard to check if these conditions apply. Countries willing to balance privacy and public health adequately should develop similar regulations to that of the European Union.

## Equity

Equity concerns about immunity certificates should not be disregarded. Of all the foreseen ways to obtain these certificates, only natural immunization resulting from having had COVID-19 does not involve any direct economic investment. On the contrary, the cost of access to the tests, which would have to be carried out continuously, could be prohibitive for many people (and also for some governments). As Baylis and Kofler (2020) wrote, “fair access to immunity testing will be a challenge for the poor and already vulnerable—low-income hourly workers, immigrants, people of color, older people, people with disabilities, people with addictions, and those who are incarcerated” (Baylis and Kofler 2020). Vaccines, on the other hand, could be expensive for those who do not benefit from public subsidies because they do not have a legal residence document, for example. Therefore, it could be plausible that some citizens choose to become deliberately infected to win their passports (Brown et al. 2020; Greely 2020; Voo et al. 2020). This would clearly be unfair, not to mention its possible consequences in terms of spread prevention because many migrants are at increased risk of contagion (Clark et al. 2020).

These risks, however, could be mitigated in various ways. First, by financing the vaccination of all the inhabitants of a country, regardless of their specific administrative and migratory situation (Bailey 2020). Secondly, by financing the tests at least for those who cannot be vaccinated for medical reasons or who have failed to achieve immunization despite being inoculated. It could even be pointed out that the tests should be subsidized for those who simply do not wish to be vaccinated. In this way, their vaccine refusal would be scrupulously respected. However, this last possibility is debatable. In the end, a vaccine refuser’s position is of little solidarity with respect to others. Therefore, it would not necessarily be unfair that those who reject vaccination should have to pay for the tests entirely from their own pocket if they want a green passport.

Last but not least, we should ensure that both certificates in digital and paper form are available. Many people who are older, poorer, or less educated may not have electronic devices that would enable them to get digital certificates. Therefore, if the legislature does not provide them with the means to bridge such technological gap, they would remain unfairly excluded. In this sense, states could follow the path traced by New York state’s Excelsior Pass, which is easily printable (even though citizens should have access to a computer in order to print it).

## International Mobility and Global Fairness

The last concern is related to the prospective impact of immunity certificates and similar immunity-based licenses on international mobility from a global fairness perspective. In the countries where these documents will be enforced, entrance will be conditional on the possession of these certificates. International air travel and other types of border crossing will thus be affected by this health policy (de Miguel Beriain and Rueda 2021). In this context, equity-related worries are not only restricted to how a citizen within a particular country has a fair opportunity to achieve the green pass or an immunity passport, but also how these public health strategies can put other citizens of less developed countries at a disadvantage. In this regard, two obstacles hinder fair international mobility: the great disparity of public health policies or governmental responses to COVID-19, and the prioritization of the interests of national or supra-regional entities (e.g., European Union) over global justice aspirations.

On the one hand, governmental responses to the COVID-19 pandemic have been far from homogeneous in the international community—and often not even within some countries— due to the divergent measures between local, regional, and national administrations. In the face of tackling the sanitary and socioeconomic crisis, the pandemic politics of individual countries have been conditioned by factors such as the regime type, its formal political institutions, and the capacities of each state (Greer et al. 2020). This diversity should be considered when evaluating the future outcomes of immunity certificates in international mobility from a global justice viewpoint. Although, indeed, vaccine entry requirements are primarily a phenomenon of global south countries, the emergence of COVID-19 has prompted richer countries of the global north to study the need for implementing travel restrictions (such as immunity passports) that are less disruptive or coercive than quarantines upon arrival or complete travel bans (Vanderslott and Marks 2021). This tendency faces the challenge of how to provide global access to these tools across different countries. While the EU Digital COVID Certificate is undoubtedly useful to give a uniform standardization of immunity passports through the European Union countries, other global agreements should be envisioned to distribute the health and freedom benefits of these certificates more fairly across the globe.

On the other hand, current public health policies are also conditioned by vested interests around socioeconomic recovery. Immunity certificates have not only the potential for making travel safe again, but they also promise the recovery and maintenance of economic activities, such as tourism, that are of significant importance for some countries and social collectives. Similarly, another not strictly health-related objective of vaccination programs is to restore the “old normality.” Having said that, economic interests are precisely one of the reasons that may explain the controversial phenomenon of vaccine nationalism.

Vaccine nationalism refers to the prioritization of domestic interests (especially of rich countries) regarding the purchase and hoarding of supplies of vaccines against COVID-19 (Lagman 2021). This tendency towards national partiality hampers a fair global vaccine allocation (Emanuel et al. 2020). Moreover, vaccine nationalism may impact the global (un)fairness of immunity passports. Vaccination is a cost-effective manner to give the majority of the population access to immunity passports. Consequently, countries that now have better access to vaccines can offer more possibilities to guarantee the widespread distribution of immunity-based licenses. The diverse immunization coverage, unequal vaccine supplies, or divergent manufacturing capacity across countries are factors that therefore may have an impact on the global fair distribution of immunity passports.

In consequence, at the moment, the unfair global distribution of access to international mobility remains one of the biggest complications with immunity certificates. This is not an insurmountable problem, though. The establishment of these certificates is a valuable step that may pave the way to a uniform international certification about one’s individual immunity status regarding COVID-19—similar to the International Certificate of Vaccination or Prophylaxis also known as the Yellow Card or Carte Jaune, created by the WHO. Furthermore, the potential certification of the immunity certificates is not the unique benefit that vaccination provides, but just an additional one. The global unfairness of the self-interested national policies conducted by most rich countries regarding the unbalanced global acquisition of vaccine supplies is outrageous mainly because vaccines are, for some people, life-saving resources. Individuals are the ultimate recipients of vaccines, not the states (Emanuel et al. 2020). In a similar vein, Jeremy Farrar has argued that vaccines should be made available to other countries as soon as the most vulnerable have been vaccinated (Looi 2021). The unfair global distribution of immunity certificates, consequently, is just a supplementary problem raised by vaccine nationalism. Besides, this problem could be mitigated. Lifting patents, international consortiums such as COVAX, and the technology transfer of manufacturing capacities (for the production of inexpensive diagnostic tests and vaccines) are desirable practices of global solidarity that might be necessary for the fair worldwide distribution of immunity certificates and of consequent international mobility. Furthermore, developed countries could perfectly tackle international inequity by providing free tests prior to boarding to travellers coming from countries where vaccines were not available, so they could obtain the immunity certificates that allow their mobility.

## Conclusion

Our analysis leads us to claim that immunity certificates can be an acceptable tool from an ethical and legal point of view if they meet certain conditions: they should not process personal data beyond what is strictly necessary, equal access to them should be guaranteed, and they should not restrict people’s autonomy to access places where contagion is unlikely. In all cases where these conditions would not apply, their use should be avoided. For instance, if states could guarantee the accessibility of tests only for those who are willing to visit a relative in a hospital, then certificates should only be requested in the hospitals. If European Union member states, for instance, could only afford tests for the citizens of a few foreign states with no access to vaccination, then certificates should only be used in those travels.

Furthermore, its utility will depend on our capability to develop tools that are able to provide us with evidence about the contagiousness of the people. If vaccines provide some kind of sterilizing immunity, that would be easier to do. If this is not the case, cheap and accessible tests would be the only way to ensure the equitable implementation of digital green certificates. Moreover, we have shown that the fair global distribution of this kind of immunity certificate remains a prime challenge that should be addressed with future global policies.

Last but not least, further research should focus on the best strategies to perform public accounting and third-party auditing of the impact of immunity certificates. Ethical governance requires monitoring the implementation and development of these tools in regard to their effective consequences on human rights and global justice. The potential of immunity-based certificates to safeguard public health while guaranteeing safe mobility and incentivizing vaccine uptake (Mills and Rüttenauer 2022) should not obscure the need for researching future side-effects of this policy. Certainly, immunity certificates will solve various problems related to current travel restrictions and may help to mitigate some of the most deleterious socioeconomic consequences of the pandemic, but they will probably create new inadvertent ones. The anticipatory study of their foreseeable consequences should be a priority during the next few months from the ethical management of this extraordinary public health policy raised by the COVID-19 pandemic.
